# Evaluation of lip diseases in peddlers on two beaches of Guarujá/Brazil: An observational study

**DOI:** 10.1590/0103-6440202406044

**Published:** 2024-12-06

**Authors:** Caio Vinicius G. Roman-Torres, Edson Gracia, Angelica Castro Pimentel, Humberto Oswaldo, Wilson Roberto Sendyk, Luana Campos

**Affiliations:** 1 Department Dentistry, University Santo Amaro, São Paulo, SP, Brazil; 2 Department of Stomatology, Federal University of Parana, Curitiba, PR, Brazil

**Keywords:** Lip, Actinic Cheilitis, Ultraviolet Rays, Sun Protection Factor

## Abstract

Sun exposure is responsible for most lip lesions and, when diagnosed late, the prognosis is unfavorable. Treatments are usually mutilating, palliative, and expensive. This study aimed to evaluate the prevalence of lip diseases and factors associated with its development in peddlers on two beaches of the Guarujá/Brazil coast. Data were collected by clinical examination and use of a questionnaire of 182 peddlers on the beaches. They also received an explanatory folder with information about oral care, and the importance of the use of sunscreen for cancer prevention. All workers diagnosed with lip injuries were referred for dental treatment. The prevalence of lip alterations was 40.7% (n = 70), furthermore, smokers included 27.5%, and alcohol consumption was 20.3%. Regarding selfie protection, 72% of workers reported wearing a hat or cap frequently, 48.4% reported using body sunscreen, and only 11.5% reported using lip balm. There were no significant differences in factors such as alcoholism and smoking related to lip lesions diagnosis. However, when characterizing these factors, 48.6% of those who drink, or smoke have lip alterations. It can be concluded that the population of peddlers on the beaches of Guarujá has a high rate of lip alteration which suggests the need to implement educational health strategies aimed at informing the population about risk factors and preventive measures for lip diseases.

## Resumo

A exposição solar é responsável pela maior parte das doenças labiais e, quando diagnosticadas tardiamente, o prognóstico é desfavorável, e os tratamentos costumam ser mutilantes, paliativos e onerosos. Este estudo teve como objetivo avaliar a prevalência de doenças labiais e os fatores associados ao seu desenvolvimento em vendedores ambulantes de duas praias do litoral do Guarujá/Brasil. Os dados foram coletados por meio de exame clínico e aplicação de questionário a 182 trabalhadores. Eles também receberam um folder explicativo com informações sobre cuidados bucais e a importância do uso do protetor solar na prevenção do câncer labial. Todos os trabalhadores com diagnóstico de lesão labial foram encaminhados para tratamento odontológico. A prevalência de alterações labiais foi de 40,7% (n = 70); além disso, os fumantes incluíram 27,5% e o consumo de álcool prevaleceu em 20,3% dos participantes. Quanto à proteção individual, 72% dos trabalhadores relataram usar chapéu ou boné com frequência, 48,4% relataram usar protetor solar corporal e apenas 11,5% relataram usar protetor labial. Não houve diferenças significativas em fatores como alcoolismo e tabagismo para o diagnóstico de lesão labial. Porém, ao caracterizar esses fatores, 48,6% dos participantes que bebem ou fumam apresentaram alterações labiais. Pode-se concluir que a população de vendedores ambulantes das praias do Guarujá apresenta alto índice de alteração labial, o que sugere a necessidade de implementação de estratégias educativas em saúde que visem orientar a população sobre os fatores de risco e medidas preventivas das lesões labiais.

## Introduction

The main change in the lip is the development of a persistent sore, lesion, lump, or ulcer that doesn't heal within a few weeks. Other signs of lip alterations may include red or white patches on the lips, numbness, tingling, a feeling of fullness in the lips, difficulty moving the lips, and swelling or thickening of the lips. Any unusual or persistent changes in the lips should be evaluated by a healthcare professional, as they could potentially indicate cancer or another serious condition ^1^ ^,^ ^2^ ^,^ ^3^.

Actinic cheilitis (AC) is a lesion with malignancy potential described as a degenerative condition of the mucosal epithelium caused by the cumulative effects of ultraviolet (UV) solar radiation and is responsible for 95% of cases of squamous cell carcinoma (SCC) of the lip ^4^. Worldwide, 529,500 people develop lip, oral cavity, and pharyngeal cancers each year, representing 3.8% of all cancer cases, and the number of cases is predicted to increase by 62% to 856,000 by 2035 due to demographic changes ^1^. In Brazil, a 2019 estimate from INCA projects 14,700 new cases of oral cancer, being the fifth most common type of cancer in men in the country. It should be noted that lip cancer is included in this estimate along with other oral cancers ^2^ ^,^ ^5^.

The classic causes of oral cancer include smoking and alcohol consumption but are also other risks factors and cofactors such as HPV, which is responsible for 20% of oral cancer cases in Brazil, with studies showing that young people are among the most affected group ^1^ ^,^ ^5^, and sun exposure. AC is most common in white men over 40 years of age with a history of chronic exposure to sunlight and artificial UV radiation ^4^ ^,^ ^6^. Among the risk groups for the disease, the peddlers on the beaches in Brazil stand out because of their direct or indirect daily sun exposure ^6^.

The main clinical symptoms of AC include dryness, atrophy, white plaques, and ulcerative lesions. In lip SCC, crusty and irregular lesions may be observed, with variable invasion into the underlying muscle ^7^. When diagnosed late, the prognosis is unfavorable, and treatments are often mutilating, palliative, and expensive for hospitals and dental care.

Given the lack of guidance, associated with chronic exposure to solar radiation and the lack of photoprotective measures, such as the appropriate use of sunscreens and hats, these workers are more likely to develop lip lesions, including AC and SCC, and are often diagnosed late ^4^ ^,^ ^6^. Due to the social importance and the limited number of studies addressing this type of approach and analysis, the objective of this investigation was to evaluate the prevalence of lip lesions among peddlers on two distinct beaches situated along the Guarujá/Brazil coastline.

## Materials and Methods

The study followed Helsinki Norms, and individuals who agreed to participate in the study signed the Free and Informed Consent Form, which was previously reviewed and approved by the UNISA Human Research Ethics Committee with CAAE: 51627021.4.0000.0081.

Four calibrated dentists were divided into seven teams, distributed about the coastal sections of Pitangueiras and Astúrias beaches, which together span 7 km long. The calibration included identifying all lip alterations, and in the three training sessions, 5 images of lesions were projected, and participants had to write down what they were. The calibration results, measured by the kappa coefficient, ranged from 0.87 to 1.00. The following quality assurance factors were considered during the training: Dryness, atrophy, white plaque, and ulcer For lip lesions, any participant with one or more alterations was considered.

The estimated number of participants was 405, based on the local city hall records, including 295 peddlers working at Pitangueiras and 110 at Asturias ^8^. Based on the prevalence of AC, the minimal sample size was calculated as 43.24%. A total of 210 workers were approached, however, 28 individuals refused to participate in the study. Thus, the sample consisted of 182 participants.

All types of peddlers on the beaches were approached, including those with fixed carts along the boardwalk, those with stationary carts on the beach, and those pushing carts along the boardwalk. Was applied questionnaire addressed sociodemographic independent variables (sex, age, ethnicity, and individual monthly income), occupational information, and health data (daily, weekly, and cumulative sun exposure, use of photoprotective measures, habits such as smoking and alcohol drinking, oral hygiene habits such as tooth brushing and use of mouthwash with or without alcohol were recorded). After completing the questionnaire, two dentists (team) examined the lip mucosa of the participants, recording findings such as atrophy, dryness, ulcers, crusts, and white or red coating. Photographs of the lesions were obtained with the camera of an iPhone® mobile (Apple, USA).

An informational brochure was prepared containing details about lip care and self-examination, such as the role of disease prevention using lip protectors and self-examination of the lips, as a nonhealing lesion in this area can be easily located and monitored for progression, as well as information about the campaign and partners. At the end, the patients diagnosed with lip lesions were referred to the Stomatology service of the Dental Specialties of Guarujá City Hall.

Data were subjected to statistical analysis, with a significance level set at 0.05. Nonparametric statistical tests were employed, and the normality of the main quantitative outcome variables was assessed using the Kolmogorov-Smirnov test (N≥30), which indicated that the variables did not follow a normal distribution.

Qualitative factors were characterized by relative frequency analysis (percentages or prevalence) using the two-proportion equality test. Bivariate analyses were conducted according to the four main outcome variables: dryness, atrophic, white plaque, and lip changes. The Mann-Whitney test was used for comparisons involving quantitative factors. Each of the main outcomes was compared with qualitative factors by distributing relative frequencies (percentages), using the chi-square test.

For each outcome, multivariate analysis was performed, that is, a multivariate logistic regression model was used to try to find a model that could predict the probability of occurrence of each lip change. Bivariate analyses were used to select the independent variables (referred to as explanatory), and all variables with a p-value < 0.2 were selected for the respective models. Finally, two statistics were used to assess the quality of the models. The Nagelkerke-R2 (also called pseudo-R2) evaluates the quality of prediction, and the Hosmer and Lemeshow test evaluates whether the model is adherent.

## Results

Among the 182 peddlers examined, 54 (29.67%) were female and 128 (70.32%) were male, with a mean age of 35.3 years and a higher prevalence of white ethnicity (44.5%). Regarding lifestyle habits, tobacco and alcohol consumption was reported by 27.5% and 20.3% of participants, respectively (Table 1).

**Table 1:** Distribution of demographic and general qualitative factors.

		N	%	P-value*
Gender	Female	54	29,7%	<0,001
	Male	128	70,3%	
Ethnicity	White	81	44,5%	Ref.
	Brown	53	29,1%	0,002
	Black	48	26,4%	<0,001
Smoking	No	132	72,5%	<0,001
	Yes	50	27,5%	
Alcoholism	No	145	79,7%	<0,001
	Yes	37	20,3%	
Exposure	Direct	131	72,0%	<0,001
	Indirect	51	28,0%	
Protection orientation	No	137	75,3%	<0,001
	Yes	45	24,7%	
Lip balm	No	161	88,5%	<0,001
	Yes	21	11,5%	
Sunscreen	No	94	51,6%	0,529
	Yes	88	48,4%	
Cap or hat	No	51	28,0%	<0,001
	Yes	131	72,0%	
	Yes	74	40,7%	

*Statistically significant p-values of 0.05 or less are highlighted in bold.

**Figure 1:**
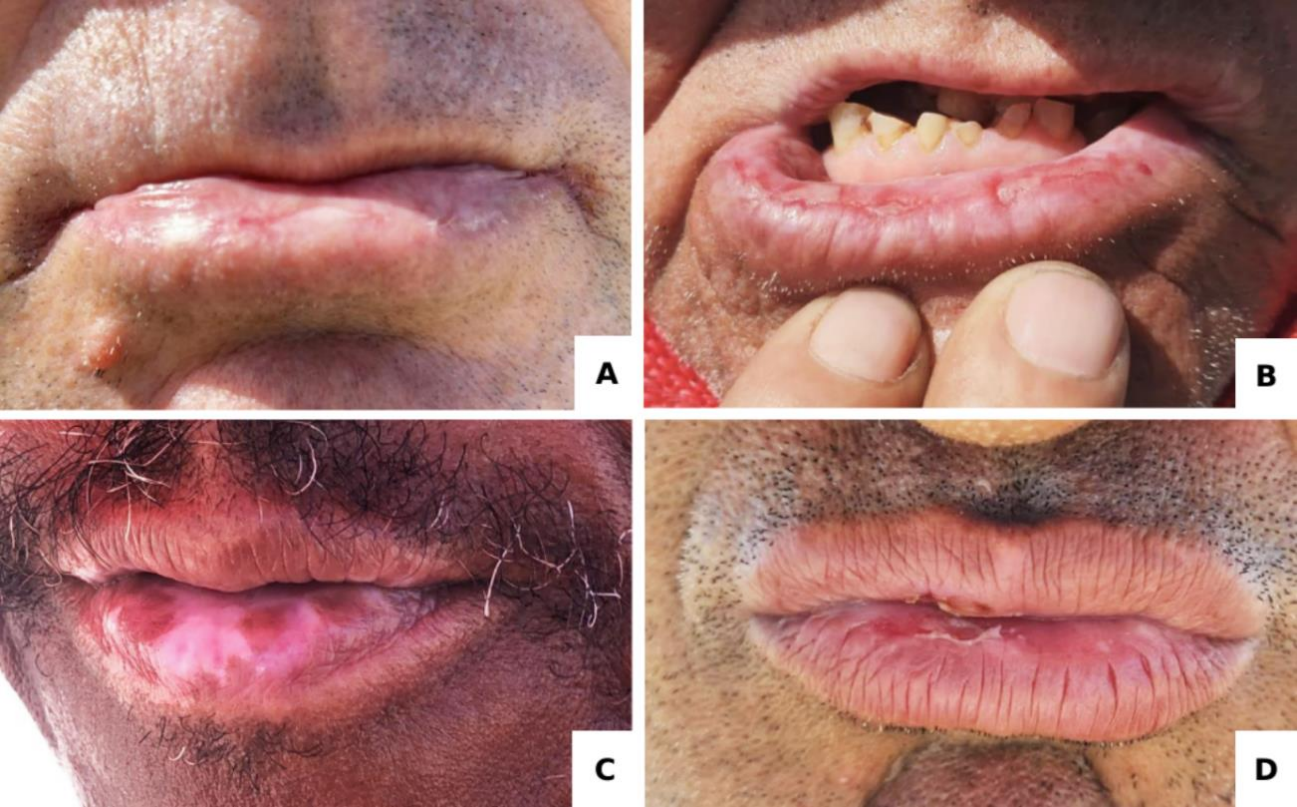
Clinical features observed lip diseases. (A) lip dryness, (B) confluent ulcers on the lower lip, (C) color change, (D) erosion on the upper and lower lip.

In terms of sun protection, most participants (72%) reported wearing a hat or cap, 48.4% reported using body sunscreen, and only 11.5% reported using lip balm. The prevalence of lip diseases was 40.7% (n=70), with 68 workers exhibiting dryness, 13 showing atrophy, 11 with white patches, and 2 with ulcerative lesions. In total, 74 participants had one or more lip alterations (Tables 1 and 2, Figure 1).

**Table 2:** Clinical aspects of lip alterations.

		N	%	P-value*
Dry Lip	No	114	62,6%	<0,001
	Yes	68	37,4%	
Atrophic	No	169	92,9%	<0,001
	Yes	13	7,1%	
Ulcer	No	180	98,9%	<0,001
	Yes	2	1,1%	
White patches	No	171	94,0%	<0,001
	Yes	11	6,0%	
Lip Changes	No	108	59,3%	<0,001
	Yes	74	40,7%	

*Statistically significant p-values of 0.05 or less are highlighted in bold.

When examining the relationship between the presence of lip diseases and qualitative factors, it was observed that male gender and direct sun exposure were statistically significant (p<0,001) (Table 3).

**Table 3:** Relationship of the presence of lip diseases with the distribution of qualitative factors.

		No		Yes		Total		P-value*
		N	%	N	%	N	%	
Alcoholism	No	86	79,6%	59	79,7%	145	79,7%	0,987
	Yes	22	20,4%	15	20,3%	37	20,3%	
Exposure	Direct	69	63,9%	62	83,8%	131	72,0%	0,003
	Indirect	39	36,1%	12	16,2%	51	28,0%	
Gender	Female	41	38,0%	13	17,6%	54	29,7%	0,003
	Male	67	62,0%	61	82,4%	128	70,3%	
Sunscreen	No	53	49,1%	41	55,4%	94	51,6%	0,401
	Yes	55	50,9%	33	44,6%	88	48,4%	
Lip Balm	No	93	86,1%	68	91,9%	161	88,5%	0,231
	Yes	15	13,9%	6	8,1%	21	11,5%	
Smoking	No	83	76,9%	49	66,2%	132	72,5%	0,114
	Yes	25	23,1%	25	33,8%	50	27,5%	
Ethnicity	White	47	43,5%	34	45,9%	81	44,5%	0,122
	Brown	37	34,3%	16	21,6%	53	29,1%	
	Black	24	22,2%	24	32,4%	48	26,4%	
Total		108		74		182		

*Statistically significant p-values of 0.05 or less are highlighted in bold.

Although statistical analyses did not shown a direct relationship between smoking and alcohol consumption with lip diseases, it was possible to identify that 48.6% of workers who drink, or smoke have either type of lip disease. When OR was calculated for these factors, it was found that drinking or smoking only are risk factors, with OR of 1.82 and 1.69, respectively (Table 4).

**Table 4:** Odds ratio of factors for lip disease.

		With Changes	No Changes	Odds Ratio
Drink and Smoke	Yes	4	10	0,56 (0,17 a 1,86)
	No	70	98	
Drink or Smoke	Yes	36	37	1,82 (0,99 a 3,33)
	No	38	71	
Only Drink	Yes	15	22	0,99 (0,48 a 2,07)
	No	59	86	
Only Smoke	Yes	25	25	1,69 (0,88 a 3,27)
	No	49	83	

According to logistic regression models, in ENTER and STEPWISE, where the coef. (B) indicates whether the factor is protective or risk increasing, p - the significance level, and OR also whether the factor is protective or risk increasing. In the ENTER method, all variables are included in the model, regardless of whether they are significant in a multivariate manner. Using the STEPWISE method, we included the same variables as the ENTER method, but the model tests them one by one and include/exclude those that are significant or no longer significant in different ways. Therefore, using the STEPWISE method, we only had variables with statistical significance (in this method, the significance for entering the model is 0.05, and for leaving it is 0.10). According to Table 5, in both models, years of work, direct exposure and male sex are risk-increasing factors, as they have an OR of more than 1.00.

**Table 5:** Logistic regression model for “Lip Diseases”. The column with coefficients (Coef. (B)) shows whether the factor/variable is risk-exposing (when the value is positive) or protective (when the value is negative). The p-value indicates the significance. The Odds Ratio (OR) greater than 1.00 indicates a risk exposing factor and when less than 1.00 it indicates a protective factor.

	ENTER					STEPWISE				
	Coef. (B)	P-value	Odds Ratio			Coef. (B)	P-value	Odds Ratio		
			OR	Inferior Limit	Upper Limit			OR	Inferior Limit	Upper Limit
Constant	-0,902	0,601				-0,373	0,485			
Age	0,022	0,290	1,02	0,98	1,07					
Working (years)	-0,094	<0,001	0,91	0,86	0,96	-0,087	0,001	0,92	0,87	0,96
Last appointment (years)	0,013	0,868	1,01	0,87	1,18					
Smoking	0,136	0,743	1,15	0,51	2,58					
Exposure (Direct)	1,031	0,013	2,80	1,25	6,31	1,048	0,009	2,85	1,30	6,27
Gender (Male)	1,280	0,008	3,60	1,39	9,31	1,336	0,001	3,80	1,67	8,64
Ethnicity (White)	-0,313	0,486	0,73	0,30	1,76					
Ethnicity (Brown)	-1,120	0,029	0,33	0,12	0,89	-0,884	0,027	0,41	0,19	0,90

*Statistically significant p-values of 0.05 or less are highlighted in bold.

## Discussion

The hypothesis that peddlers on the beaches in Guarujá, Brazil have high rates of lip diseases was confirmed, with a prevalence of 40.7% based on clinical examination. This data is highly relevant, as there were no previous scientific studies specifically addressing this at-risk population ^9^ ^,^ ^10^.

Regarding other Brazilian beach workers, the literature supports the findings of this study. Silva et al (2006) ^11^ observed that more than 43% of fishermen in Florianopolis, Brazil were aware of the potential malignant transformation of their lip lesions. Similarly, Lucena et al. (2012) identified actinic cheilitis (AC) in more than 15% of workers at five urban beaches in Natal, Brazil ^6^.

A common issue highlighted is the socioeconomic characteristics that revealed the minimal importance placed on self-care, harmful life and health habits, in addition to unawareness of lip diseases. In a previous Brazilian study, the authors showed that individuals with less schooling and lower economic levels make fewer visits to the dentist; as such, they do not have the opportunity to address these problems individually during dental appointments ^12^.

In this study, no positive association was found between smoking and alcohol consumption and the rate of lip diseases. However, the frequency of workers with lip alterations who smoked or drank was 48.6% and 33.8% for smoking only. This corresponds to an odds ratio of 1.82 for "drinking or smoking" and 1.69 for "smoking only. Thus, the frequency of alterations in the group of subjects who drank or smoked only was high, which is consistent with other studies ^4^ ^,^ ^6^.

In line with the literature, most workers considered wearing a hat or cap as a form of protection ^5^ ^,^ ^6^. In the present study, this number was even higher, where 72% believed they were protected. Nevertheless, they believe that are protected against sun radiation, disregarding the importance of using sunscreen and lip protectors, whose use was observed in only 48.4% and 11.5% of participants. This negligence reflects the lack of knowledge on the biggest part of workers about the harmful effects of sun radiation, demonstrating the importance of the need to introduce preventive measures for this population.

As expected, this study showed that most participants affected by lip diseases were men. This can be attributed to cultural factors, as men do not always take care of themselves as well as women, as well as, in tropical countries such as Brazil, where many men work in outdoor occupations or have a habit of prolonged exposure to sun radiation, these lesions are more common due to high sun exposure and inadequate use of protective products such as sunscreens and hats ^2^ ^,^ ^4^ ^,^ ^6^ ^,^ ^7^ ^,^ ^9^.

It’s well known that prolonged sun exposure is an important risk factor for the development of the most feared lip diseases, such as lip cancer and AC, which are characterized by ulcerations, atrophy, and/or leuko and/or erythroplastic areas ^2^ ^,^ ^3^. The lips are more susceptible to sun radiation because of their thin epithelial thickness and keratin layer, lower melanin protection, and sparse secretion from the sebaceous and sweat glands ^13^. This susceptibility is consistent with the data obtained in this study, which showed a significant correlation between direct sun exposure and the presence of lip injuries among workers.

Despite some limitations of this study-including the lack of information from the surveyed population, working in pressure from the supervisors at the busiest work time, as well as, the lack of interest from persons with lip diseases to visit the Center for Dental Specialties, even after orientation by the professionals involved in the action; this research may contribute to a deeper understanding on the health professionals and authorities regarding the epidemiological aspects of lip diseases in this population.

In conclusion, the population of peddlers on the beaches of Guarujá/ SP has a high rate of lip alterations, being important to intensify educational and preventive measures, as well as ensure suitable curative actions for this population.
